# Somatic symptom disorders and utilization of health services among Palestinian primary health care attendees: a cross-sectional study

**DOI:** 10.1186/s12913-021-06671-2

**Published:** 2021-06-29

**Authors:** Zaher Nazzal, Beesan Maraqa, Marah Abu Zant, Layali Qaddoumi, Rana Abdallah

**Affiliations:** 1 Department of Medicine, Faculty of Medicine and Health Sciences, An-Najah National University, Nablus, Palestine; 2 Primary Healthcare Directorate, Palestinian Ministry of Health, Ramallah, Palestine

**Keywords:** Somatic symptom disorder, Primary care clients, Risk factor, Palestine

## Abstract

**Background:**

Many primary health care (PHC) clients come in with medically unexplained complaints, leading to frequent consultations and high usage of services and healthcare costs. This study aimed to determine the prevalence of somatic symptom disorder (SSD) among PHC attendees and explore its relation to other mental conditions and risk factors.

**Methods:**

A cross-sectional design was used to interview 400 attendees. Men and women aged over 18 years old without a psychiatric diagnosis were invited to participate. The Somatization scale of the Four-Dimensional Symptom Questionnaire was used to assess somatic symptom disorders. It is a valid tool to be used in a PHC setting. We used the Chi-square test and multivariable logistic regression to explore determinant variables.

**Results:**

Prevalence of SSD was 32.5% (95%CI = 27.9–37.1%). The most common symptoms were painful muscles (61.5%) followed by back pain (52.3%). Female gender [adjusted OR = 2.1 (95% CI = 1.2–3.7)], chronic diseases [adjusted OR = 2.4 (95%CI = 1.3–4.5)], depression [adjusted OR = 3.3 (95%CI = 2.0–5.5)], and anxiety [adjusted OR = 2.1 (95%CI = 1.2–3.6)] were all associated with SSD. In addition, frequent primary health care attendance was found to be associated with SSD [adjusted OR = 2.4 (95%CI = 1.4–4.1)].

**Conclusions:**

SSD significantly higher among females, patients with chronic diseases, clients with anxiety and depressive disorders, and patients with frequent doctors’ visits. Painful muscles and back pain are the most common symptom presented by patients, and this could be used initially by PHC physicians as a signal to consider for screening.

**Supplementary Information:**

The online version contains supplementary material available at 10.1186/s12913-021-06671-2.

## Introduction

Somatization is the expression of psychological or emotional distress through physical symptoms that are otherwise unexplained. It has been argued that it is so common in primary health care (PHC) as to be considered the norm, not the exception [1]. Somatic symptom disorder (SSD), the new term used for somatization in the Diagnostic Statistical Manual 5th edition (DSM-V), requires significant attention to physical symptoms being experienced by a person such as pain, weakness, or shortness of breath that contributes to discomfort and/or functioning problems. The person has physical symptoms related to repetitive thinking, feelings, and behaviors. Physical symptoms may or may not be associated with a particular medical condition, but people experience symptoms and feel ill [2].

SSD has been considered as a significant problem, particularly in PHC [3]. Patients with numerous recurring physical symptoms that do not appear to have any apparent biological basis are frequent among PHC clients [4]. The prevalence of SSD is inconsistent and varies significantly between different countries and communities, depending on the population of the underlying study and the diagnostic criteria used in single studies [5–7]. The results of a regional Iranian study showed a high prevalence of somatic symptoms of varying severity. Age, marital status, education, socio-economic status, anxiety, and depression were reported as significant risk factors [6]. On the other hand, SSD was associated with female gender and low educational level among Kuwaiti PHC attendees [5].

The link between SSD and other mental health conditions, such as depression and anxiety, has been extensively studied. A large population-based study showed a strong relationship between depressive and anxiety disorders and somatic symptoms [8]. A further general population study showed that somatic symptoms and anxiety are often overlapping and that they are associated with the increased use of healthcare services [9]. Somatic symptoms were common among the younger population, and an association with depression was reported. The combination of somatic, anxiety and depression syndrome described as the ‘somatization-anxiety-depression (SAD) triad has been reported to be a significant disease burden [7, 10], as well as being associated with greater severity and duration of depression and psychiatric co-morbidity and a strong correlation to suicidal plans and attempts [11].

SSD is mainly present in PHC settings; it is linked to patients’ frequent visits to clinics, contributing to recurrent use of medical services and frustration for patient and doctor alike [3]. In compared to healthy individuals, SSD patients had a worse quality of life (QOL). Additionally, SSD patients had worse general and family functioning than healthy persons [12]. The attendance level of such patients was calculated to be 60% higher than that of non-diagnosed patients [7]. It increases the use of existing services and the burden on doctors and health staff, particularly in low and medium incomes countries like Palestine, which suffers from occupation and scarcity of resources.

In Palestine, a significant number of patients with unexplained medical symptoms visit PHC clinics daily, despite clinical training improvements for primary care physicians aimed at early detection of mental health conditions. Most patients go unnoticed in the context of a lack of a screening method to be applied at the primary care level for somatic symptom disorder. To the best of our understanding, the SSD studies in Palestine are minimal, and diagnostic risk factors have not been identified. Given the seriousness of the problem and the associated diagnosis of other mental disorders, such as depression and anxiety, we conducted this study to determine the prevalence of SSD, factors connected to it, and the relation between this disorder and anxiety and depression.

## Materials and methods

### Study design and population

A cross-sectional study was conducted in large PHC centers in North Palestine. We selected the sample through the use of convenient sampling methods, which included a serial recruitment of participants attending primary health care clinics in Nablus district, the second most populous district in the West Bank. The study had no age or gender restrictions. Anyone seeking medical attention at the designated centers was contacted and informed of the study’s objectives, as well as asked for permission to participate in the study and complete the questionnaire. Visits to the clinic were for general medical problems and/or regular wellness visits, and the interviews took place between July and Jan 2020. The majority of Palestinian communities living in the West Bank are covered by national health insurance, including all PHC services. Legibility criteria for inclusion in the study are any person –male or female- above 18 years of old with no previous mental health illness diagnosis.

The data collection was carried out by three trained researchers (family physicians), who completed the questionnaires after conducting interviews with the participants and recording their responses. To ensure quality, the researchers were trained in interviewing techniques, as well as how to build rapport and trust. Because the participant is frequently required to provide sensitive and personal information to the interviewer directly, privacy and confidentiality were specified from the start. The interviewees were assured that their participation and responses would have no impact on the care they receive or their relationship with their providers. Participants who did not complete the survey through the interview were excluded, as were those who had too many missing values.

The sample size was calculated with the assumption that the prevalence of somatization in Palestine is comparable to levels in some eastern Mediterranean countries, which ranged from 11 to 35% [5, 13]. Given the unique Palestinian situation in the presence of occupation as an additional psychological factor, we assumed that the SSD prevalence would be 35%, with a confidence interval of 95% at the significance level of 5%, a minimum sample size of 364 would be necessary for the study objectives to be achieved.

The study was approved by the An-Najah National University institutional review board (IRB) and the Palestinian Ministry of Health. After the purpose and objectives of the research were presented, PHC clients were approached and encouraged to participate voluntarily in the study.

### Measures

The 16-point Somatization Scale of the four-Dimensional Symptom Questionnaire (4DSQ) has been used to test the SSD since it is a reliable tool used for assessing somatization in PHC [14]. The questionnaire is designed to evaluate common somatic symptoms among PHC patients and determine whether any additional diagnoses are needed. It is designed on the basis that “Average” people experiencing one or even a few scientifically unexplained signs, for example, being dizzy with stomach pain, could be a normal finding under stressful circumstances. Yet, having other unexplained encounters originating from various organ systems (i.e., stomach pain and palpitation and muscle aches) may indicate SSD [15]. The instrument is coded on a continuous scale varying between 0 and 32. Coding was used as a categorical group for the 4DSQ-somatisation questionnaire: 0–10 is considered low or absent, moderately elevated if score 10–20 and high if the score was more than 20 [14].

The other parts of the 4DSQ assessed for anxiety (12 items) and for depression (6 items). For each symptom in each scale, 0 points are recorded if a symptom is absent, 1 point if a symptom is ‘sometimes’ present, and 2 points if a symptom is ‘regularly’ or more often present. Therefore, the total 4DSQ score for anxiety ranges from 0 to 24, with scores of 0–3, 4–8, and 9–24 representing ‘low,’ ‘moderately high’ and ‘very high’ risk of anxiety level. For depression, the total 4DSQ score ranges from 0 to 12, with scores of 0–2, 3–5, and 6–12 representing ‘low,’ ‘moderately high’ and ‘very high’ risk of depression levels, respectively [14].

The 4DSQ validation process began with two independent bilingual native Arabic spokespersons translating it to Arabic. One was a medical professional and the second was an official translator (non-medical background). We then compared the two versions until we came to an agreement. Another bilingual English-to-Arabic translator, who spoke a native Arabic, translated back the Arabic version to English. Then, the research team compared the two English versions of the questionnaire. After minor linguistic changes, a meeting was then held to finalize the translated Arabic version.

Two experts in the field (family medicine and psychiatry) performed the content validity, confirming the scale items’ selection. Following that, a pilot test with 40 respondents was undertaken to verify that the research instrument was sufficiently clear and legible for respondents to understand and reply to, and the instrument’s reliability was tested; the Cronbach’s alpha was =0.85.

The frequency of doctor visits was measured via a direct question asking participants about their number of visits per month to PHC doctors. There is no standard definition for frequent doctor visits, varying from 5 to 20 visits per year [16]. In this study, doctor visits were grouped as one visit or less per month vs. more than one visit per month.

Based on a literature review [6, 17–19], the following variables were independently identified as potential risk factors for SSDs: gender, age, marital status, levels of education, chronic diseases, and symptoms of depression and anxiety Age was split into two groups, with 50 years of age as a cutoff point. The marital status of participants divided into two groups: single vs. married, widowed, or divorced. The level of education was divided into two categories (an alphabet and school or university and higher). Chronic diseases were assessed using a yes/no question subjectively reported by participants.

### Data analysis

The statistical package for social science (SPSS v. 21) software was used for data management. Descriptive statistics, including mean, SD, frequencies, and percentages, were used to describe data. SSD, depression, and anxiety were recorded as dichotomous variables like the presence of SSD (a score < 11 = 0, ≥10 = 1) or the absence of a depression (a score < 3 = 0, ≥3 = 1), or anxiety (a score < 4 = 0, ≥4 = 1). Each of them was then used as a dependent variable and assessed with background variables. Chi-square test, with odds ratios (OR) and their respective 95% confidence intervals (CI) was used to determine associations for categorical variables. To assess the independent factors associated with SSD and to adjust for confounders, we incorporated all variables that were significant in the univariable analysis into a multivariable binary logistic regression model. The significance level was set at a *p-value* of < 0.05. The performance to indicate SSD was examined using the receiver operating characteristic (ROC) curve. The curve represented a plot of sensitivity versus 1 – specificity. The area under the curve (AUC) was derived from the ROC curve.

## Results

A total of 440 PHC clients were invited to participate in the study, with 400 of them agreeing to do so and completed the interview; resulting in a 90% response rate. Almost three fourths (71.8%) were female, and more than half (52.3%) were over 50 years of age. The majority were married (77.5%), unemployed (66.2%), and had an average monthly income of 300-600JD (52.7%). Almost two thirds (64%) reported having a chronic disease such as hypertension, diabetes, or coronary artery disease, and 23.3% of the participants have more than one chronic disease at the same time. For doctor visits, 22.6% saw a doctor more than once a month. Table 1 presents the background characteristics of the sample. Almost one third (32.5%) [95%CI = 27.9–37.1%] of the participants found to have SSD, 41.8% [95%CI = 36.9–46.8%] have depressive disorders, and 38.5% [95%CI = 33.7–43.5%] have depressive disorders (Table 1).

**Table 1:** Distribution of socio-demographic characteristics of the total sample and clients with mental disorders

***Variables***	Total [*n* = 400 (100%)]	Somatic symptom disorder [*n* = 130 (32.5%)]	Depression [*n* = 167 (41.8%)]	Anxiety [*n* = 154 (38.5%)]
**Age**				
18–39 years	109(27.3%)	25(22.9%)	36 (33.0%)	47 (43.1%)
40–49 years	82 (20.5%)	33(40.2%)	32 (39.0%)	41 (50.0%)
50–59 years	107 (26.8%)	44 (41.1%)	50 (46.7%)	40 (37.4%)
≥ 60 years	102 (25.5%)	28 (27.5%)	49 (48.0%)	26 (25.5%)
***P-value***		**0.009**	***0.093***	***0.005***
**Gender**				
Male	113(28.2)	21(18.6%)	35 (31.0%)	14 (12.4%)
Female	287(71.8)	109(37.9%)	132 (46.0%)	140 (48.8%)
***P-value***		**< 0.001**	***0.006***	**< 0.001**
**Marital Status**				
Married	310(77.5%)	87(28.1%)	112 (36.1%)	111 (35.8%)
Single†	90(22.5%)	43(47.7%)	55 (61.1%)	43 (47.8%)
***P-value***		**< 0.001**	**< 0.001**	***0.40***
**Educational Level**				
School	268(67.2%)	97(36.2%)	131 (48.9%)	34 (26.0%
niversity	131(32.8%)	32(24.4%)	35 (26.7%)	119 (44.4%)
***P-value***		**0.018**	**< 0.001**	**< 0.001**
**Employed**				
No	265 (66.3%)	94 (35.5%)	118 (44.5%)	118 (44.5%)
Yes	135 (33.8%)	36 (26.7%)	49 (36.3%)	135 (26.7%)
***P-value***		***0.075***	***0.114***	***0.001***
**Monthly Income**				
< 300JD	96(24.0%)	40(43.1%)	56 (60.2%)	21 (21.9%)
300-600JD	211(52.7%)	64(30.3%)	82 (38.9%)	89 (42.2%)
> 600JD	93(23.3%)	26(27.1%)	29 (30.2%)	44 (47.3%)
***P-value***		**0.04**	**< 0.001**	**< 0.001**
**Chronic Disease**				
None	144(36%)	35 (24.6%)	50 (35.2%)	63 (44.4%)
One	163(40.7%)	62 (37.8%)	73 (44.5%)	59 (36.0%)
More than one	93(23.3%)	33 (35.1%)	44 (46.8%)	32 (34.0%)
***P-value***		**0.028**	***0.123***	***0.653***
**Doctor visit**				
≤ Once a month	309 (77.4%)	84(27.2%)	121 (39.2%)	108 (35.0%)
> Once a month	90 (22.6%)	45 (50.0%)	46 (51.1%)	45 (50.0%)
***P-value***		**< 0.001**	**0.052**	**0.010**
**Doctor visit**				
< 1 a month	71(17.8)	13(18.3)	20 (28.2%)	29 (40.0%)
1 a month	238(59.5)	71(29.8)	101 (42.4%)	79 (33.2%)
2 a month	56(14.0)	24(42.8)	26 (46.4%)	27 (48.2%
3 a month	21(5.2)	12(57.1)	12 (57.1%)	11 (52.4%)
≥ 4 a month	14(3.5)	10(71.4)	8 (57.1%)	8 (57.1%)
***P-value***		**< 0.001**	***0.053***	***0.001***
**Depression**				
Low	233(59.2%)	44 (18.9%)	*–*	*–*
High	167(41.8%)	86 (51.5%)		
***p-value***		**< 0.001**		
**Anxiety**				
Low	264 (61.5%))	87 (21.2%)	*–*	*–*
High	154 (38.5%)	77 (50.0%)		
***p-value***		**< 0.001**		

†Include unmarried, widowed, and divorced, *JD* Jordanian Dinar

Significantly higher proportions of SSD were found among 40–49 and 50–59 age groups [40.2 and 41.1%, *p*-value < 0.009], female clients [(37.9% vs 18.6%), *p-value* < 0.001], clients with single marital status [(47.7% vs 28.1%), *p-value* < 0.001], clients with school or illiterate educational level [(36.2% vs 24.4%), *p-value* = 0.018], clients with very low monthly income [(43.1% vs 30.3 and 27.1%) *p-value* = 0.04], clients with one or more than one chronic diseases [(37.8 and 35.1% vs 24.6%), *p-value* = 0.028], those with high depressive scores [(51.5% vs 18.9%), *p-value* < 0.001], and those with high anxiety scores [(66.6% vs 26.0%), *p-value* < 0.001]. SSD were significantly higher among frequent users of PHC services [(50.0% vs 27.2%), *p-value* < 0.001] (Table 1).

We conducted multivariable logistic regression to assess factors independently associated with SSD and to control for possible confounders. SSD was associated with: female gender [*p-value* = 0.043 adjusted OR = 2.1 (95% CI = 1.2–3.7)], clients with chronic diseases [*p-value* = 0.01 adjusted OR = 2.4 (95%CI = 1.3–4.5)], clients with high depression scores [*p-value* < 0.001 adjusted OR = 3.3 (95%CI = 2.0–5.5)], and clients with high anxiety scores [*p-value* = 0.031 adjusted OR = 2.1 (95%CI = 1.2–3.6)]. Additionally, patients with high SSD were found to have significantly higher frequency of doctors visit per month [*p- value* = 0.003 adjusted OR = 2.4 (95%CI = 1.4–4.1)] (Table 2).

**Table 2:** Logistic regression analysis of predictors of high somatic symptom disorder among PHC clients

***Variables***	S.E	***P*** Value	Adjusted OR	95%CI
**Age**				
18–39 years†				
40–49 years	0.387	0.164	1.7	0.80–3.7
50–59 years	0.379	0.213	1.6	0.76–3.4
≥ 60 years	0.439	0.311	0.6	0.27–1.5
**Gender**				
Male†	0.331	0.043	2.1	1.2–3.7
Female				
**Marital Status**				
Married†	0.314	0.071	1.8	0.9–3.2
Unmarried				
**Educational Level**				
School†	0.331	0.823	1.1	0.56–2.1
University				
**Salary**				
< 300JD				
300-600JD	0.36	0.55	0.81	0.40–1.6
> 600JD†	0.43	0.52	0.76	0.33–2.1
**Chronic Disease**				
No†	0.33	0.010	2.4	1.3–4.5
Yes				
**Doctor visits**				
≤ Once a month†	0.28	0.003	2.4	1.4–4.1
> Once a month				
**Depression**				
Low†	0.262	< 0.001	3.3	2.0–5.5
High				
**Anxiety**				
Low†	0.323	0.031	2.1	1.2–3.6
High				

†Reference group ***OR*** Odds Ratio ***CI*** confidence interval.

Common physical complaints were evaluated among participants and highly prevalent among clients with moderate to high SSD scores. The most common complaints were painful muscles (61.5%) and back pain (52.3%), followed by tingling in the fingers (43.8%), neck pain (40.8%), and excessive sweating (39.2%) (Table 3).

**Table 3:** Distribution of physical complaints among the participants and its relation with high somatic symptom disorder score

Complain	Total	Somatic symptom disorder		OR (95% CI)
		Low	High	
**Painful muscles**	125 (31.3%)	45 (16.7%)	80 (61.5%)	8 (4.9–12.8
**Back pain**	105 (26.3%)	37 (13.7%)	68 (52.3%)	6.9 (4.3–11.3
**Tingling fingers**	86 (21.5%)	29 (10.7%)	57 (43.8%)	6.5 (3.9–10.9)
**Neck pain**	74 (18.5%)	21 (7.8%)	53 (40.8%)	8.2 (4.6–14.4)
**Excessive sweating**	81 (20.3%)	30 (11.1%)	51 (39.2%)	5.2 (3.1–8.7)
**Bloated feeling in the abdomen**	61 (15.3)	12 (4.4%)	49 (37.7%)	13 (6.6–25.6)
**Headache**	60 (15.0%)	15 (5.6%)	45 (34.6%)	9 (4.8–16.9)
**Dizziness or feeling light-headed**	56 (14%)	17 (6.3%)	39 (30.0%)	6.5 (3.5–11.9)
**Shortness of breath**	37 (9.3%)	5 (1.9%)	32 (24.6%)	17 (66–457)
**Blurred vision or spots in front of your eyes**	39 (9.8%)	11 (4.1%)	28 (21.5%)	6.5 (3.1–13.5)
**Nausea or an upset stomach**	33 (8.3%)	6 (2.2%)	27 (20.8%)	11.5 (4.7–28.8)
**Abdominal pain or stomachache**	29 (8.3%)	3 (1.1%)	26(2.0%)	22.3 (6.6–75.1)
**Palpitations**	33 (8.3%)	8 (3.0%)	25 (19.2%)	7.8 (3.4–17.8)
**Chest pressure or a tight feeling**	25 (6.3%)	2 (0.7%)	23 (17.7%)	28 (6.7–124.3)
**Chest pain**	12 (3.5)	3 (1.1%)	9 (6.9%)	6.6 (1.6–24.9)

*Chi-squared test*

Clients with moderate to high SSD scores are more likely to suffer from physical complaints than those with low scores. They are eight times more likely to have painful muscles, 6.9 times more likely to have back pain, 6.5 times more likely to have tingling figures, 8.2 times more likely to have neck pain, and 5.2 times more likely to have excessive sweating. Table 3 presents in detail the frequency of physical complaints and compare between the two groups.

**Fig. 1:**
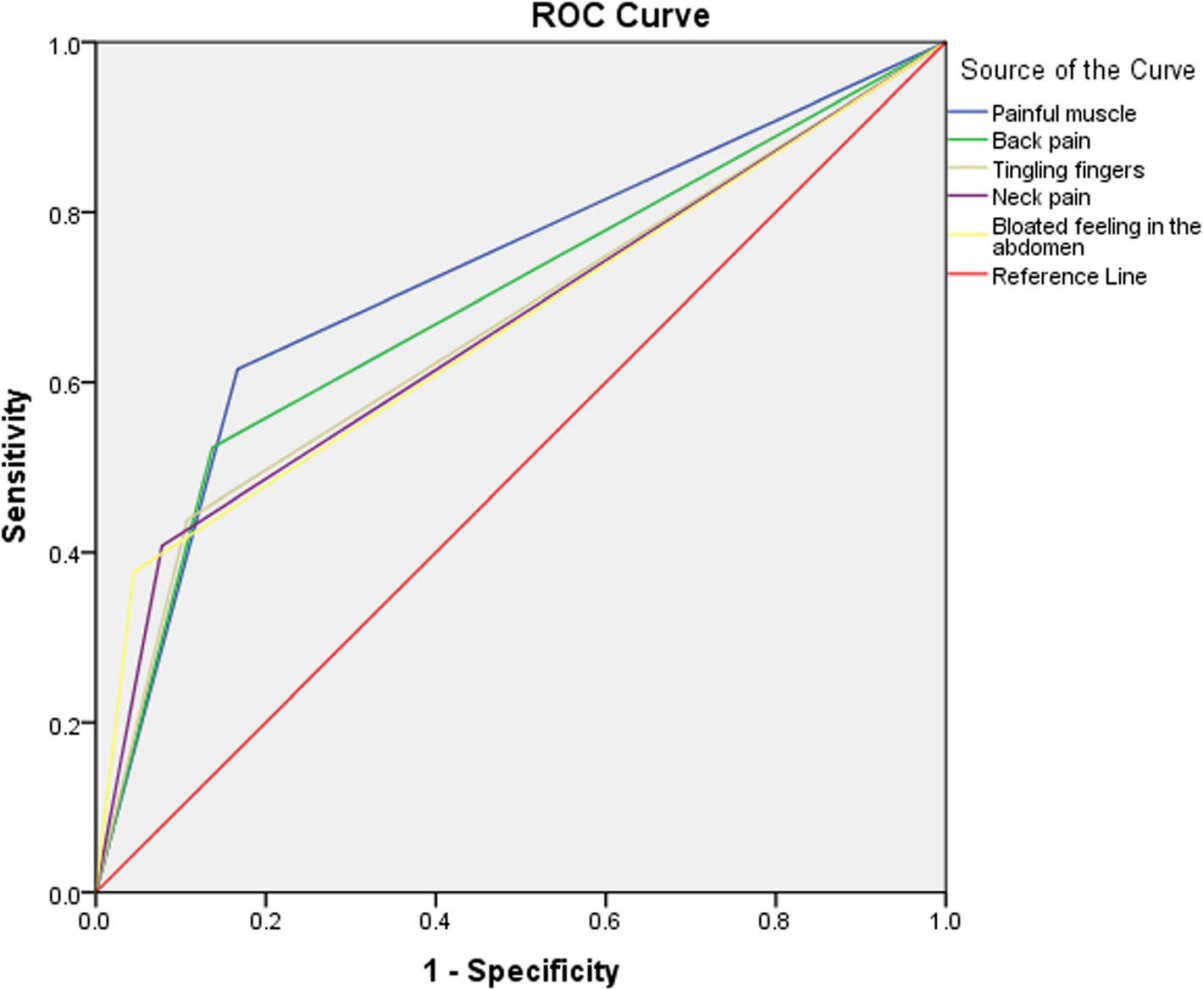
Receiver operating characteristic curve of physical complaints (painful muscle, back pain, tingling fingers, neck pain, and feeling in the abdomen) for the Somatic symptom disorder scores. The area under the curve (AUC) ranged between 0.66 and 0.72

The probability for each symptom to predict SSD was measured by ROC analysis of physical complaints. The ROC showed significant predictive power of painful muscles (AUC = 0.724), back pain (AUC = 0.693), tingling fingers (AUC = 0.666), neck pain (AUC = 0.665), and bloated feeling in the abdomen (AUC = 0.666) for high somatic symptom (SSD) score (Fig. 1).

## Discussion

Somatic symptom disorders in primary care have not been extensively researched compared with other mental disorders. The few studies found didn’t use standardized criteria for sensitive comparison and enhancing generalist care [4]. However, the present study shows that SSD is prevalent in primary care patients in Palestine, 32.5%of whom have a moderate and high somatization score. This result is in the most upper range, comparable to other studies worldwide. A systemic review reported a prevalence range from 26.2 to 34.8% of SSD diagnosable by either DSM criteria and/or ICD-10 criteria in the PHC setting [19]. Additionally, it was the most prevalent mental health morbidity among Kuwaiti primary care attendees [20]. Such statistics demonstrate that SSD presents a highly significant public health concern that is not trivial, especially in the Palestinian community with its unique conflict situation, as this will carry a higher burden on health care setting in terms of health care utilization and cost compared to other economically and politically stable countries [16].

A significant result is obtained in this study is the association between SSD with doctor visits, which reflects health care utilization. Literature showed a consensus that underdiagnosed SSD would result in higher health care utilization, higher morbidity, and lower health-related quality of life [16, 21, 22]. Patients with SSD appear to have frequent visits and contacts with their PHC physicians as they feel unhappy with their medical tests, resulting in increased use of medical resources. Patients who have mental disorders tend to be frequent visitors [23]. This, in turn, will pressure the already overloaded PHC clinics in Palestine on the one hand and increasing health care costs in this low-income country on the other side. Furthermore, it is a big challenge for PHC physicians in dealing with uncertainty regarding the diagnosis in this highly-stressed clinical population with their usually somatically focused health concepts. A situation that will result in unnecessary medical treatments and referrals whereas short term psychotherapy could be the more convenient, cheaper and best choice [24].

The association between SSD and the female gender is consistent with previous findings. Our results align with Alkhadhari and colleagues who reported a strong association between SSD and female gender among the Kuwaiti population [5]. This could be attributed to specific cultural norms related to the Arab world and the inherent differences between males and females concerning somatic and emotional perception. Gender imbalance in the rates of abuse and violence; gender disparities in the incidence of anxiety and depressive disorders; and gender inequality may also affect these findings [25].

Mechanisms linking co-morbid mental and chronic diseases are complex and bi-directional. Chronic illness can affect mental health and lead to psychological disorders, and an individual may be subjected to chronic physical disease by a psychological disorder. Other mental and physical conditions share risk factors such as chronic social stress, inactivity, overweight, smoking, alcohol use, and endocrine disorders [26]. Many studies examined the association between SSD and the presence of chronic disease. They revealed a strong association, increasing in strength, with an increasing number of chronic diseases diagnosed in a single person [20, 27]. For example, a large population-based study found a strong association between heart attacks and the history of major surgeries and SSD [18]. SSD, on the other hand, was the most common co-morbid mental disorder associated with chronic disease and the one most implicated in poorer prognosis, increased use of health care, higher cost of health care, and more inadequate compliance with treatment [20, 28, 29].

A statistically significant relationship between SSD and depressive disorder has been shown in several studies [6, 30, 31]. The overlap between these disorders was also documented in various studies [5, 13]. As a result, screening for mental health problems in patients with unexplained symptoms could be recommended based on these results. However, this was not justified by a large longitudinal study in the United Kingdom [30]. So, further studies are needed to predict anxiety and depression diagnosis among patients with multiple visits with unexplained medical symptoms.

Painful muscles and back pain are the most common somatic symptoms in our study and were significantly more frequent among clients with SDD. Similarly, back pain and tiredness were more frequently related to this condition in Turkish migrants living in Germany [32]. Stomach pain and painful leg and arm joints were more frequent associations among the Iranian population [6]. Different symptoms may be related to SSD in different groups and cultures.

SSD symptoms were generally seldom recognized by primary care providers. Only half the individuals with psychiatric illnesses had been recognized by primary care doctors as part of World Health Organization research [33]. Likewise, the low mental problem detection (11.6%) rate of general practitioners was reported in a research by primary care doctors in the Gaza Strip [34]. Despite the poor detection rate among participants, the authors in this research revealed certain aspects which contributed to make diagnosis more simpler. Factors such as the female patient with chronic illness who was reported to have a high anxiety and depression score and required multiple visits for various unexplained complaints, among others, would inherit the sensation of SSD by the primary care physician.

The study has many strong points, including novelty, using a standard questionnaire that has been designed specially to be used in PHC settings and the large sample size that could reduce sampling bias. Non-respondents and those who were unable to complete the survey owing to time constraints for a doctor’s appointment were estimated to make up less than 10% of those contacted, indicating a low non-response rate. However, some limitations should be taken into consideration as the study was based on self-reported data, which makes the reliability of the findings questionable. In order to ensure the reliability of participants’ responses, the interviews were conducted in a place that guaranteed their privacy and was administered by trained family physicians who used a standardized procedure in data collection. Secondly, given the large sample size for this study to determine the prevalence, it is likely that the sample was not powerful enough to identify substantial differences with individual determinants. Being a cross-sectional study, not longitudinal, precludes any causal association between SSD and its risk factors.

Patients with SSDs are frequently misdiagnosed and over-investigated. With this in mind, we must be fully aware of the unavoidable unintended consequences of over diagnosis, as with any screening program [35], and the possibility of missing underlying physical illness. Taking this into consideration, this problem should be addressed as an integral part of the screening program. However, once these patients have been diagnosed, they will be directed to the appropriate specialization, which will result in fewer visits to PHC.

In conclusion, SSD is prevalent in PHC settings, challenging physicians for possible diagnosis, resulting in increased utilization of healthcare resources, and increased cost. We believe that the results of this study will present an opportunity for increased awareness of mental health issues presented in PHC. It also provides an opportunity for formulating a protocol in assessing and managing these patients to reduce the burden of this condition on patients, providers, and the healthcare system. Patients with multiple visits per month could have SSD and go misdiagnosed for a long time, compromising their quality of life and functioning. Primary health care providers should have a high index of suspicion particularly for patients with several recurring unexplained symptoms. They will also be helped to increase their confidence in making accurate diagnoses by encouraging frequent training sessions.

## Supplementary Information

**Additional file 1.**

## Data Availability

The dataset supporting the conclusions of this article is included within the article and its additional file.

## Abbreviations

AUC: Area under the curve
CI: Confidence intervals
DSM-V: Diagnostic statistical manual 5th edition
4DSQ: Four-dimensional symptom questionnaire
OR: Odds ratios
PHC: Primary health care
ROC: Receiver operating characteristic
SAD: Somatization-anxiety-depression
SSD: Somatic symptom disorder
